# Intramolecular Phosphine-Promoted Knoevenagel Based Redox-Reaction

**DOI:** 10.3390/molecules27154875

**Published:** 2022-07-29

**Authors:** Niklas Feuge, Jan C. Namyslo, Dieter E. Kaufmann, René Wilhelm

**Affiliations:** Institute of Organic Chemistry, Clausthal University of Technology, 38678 Clausthal-Zellerfeld, Germany; niklas.feuge@tu-clausthal.de (N.F.); namyslo@ioc.tu-clausthal.de (J.C.N.); dieter.kaufmann@tu-clausthal.de (D.E.K.)

**Keywords:** Knoevenagel reaction, diketones, phosphines, redox-reaction, mechanism

## Abstract

A Knoevenagel based redox-reaction promoted by intramolecular phosphine sources is presented for the first time. The influence of different diketones, aldehydes, bases and acids was investigated. The effects of different substituents were evaluated based on their electronical influence on the diketone structure. With the obtained results a mechanism is proposed, giving information about transition states formed during the reaction, which can lead to different products. This type of an internal redox transformation with a phosphine oxide moiety remaining in the molecule after the redox reaction represents a new type of reaction.

## 1. Introduction

The carbon–carbon bond formation is the corner stone in the formation of many industrial compounds used in products, such as pharmaceuticals, insecticides and pesticides, and as intermediates for natural product generation [1,2,3,4,5,6].

**Scheme 1:**
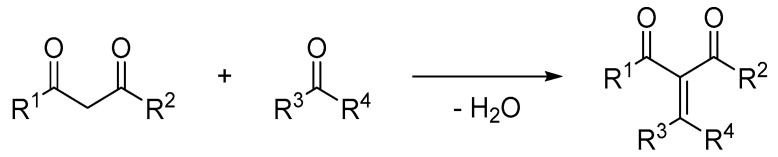
Conventional Knoevenagel condensation.

In 1894 the first reaction of formaldehyde with diethyl malonate, known as the Knoevenagel condensation, was discovered [7]. Nowadays the Knoevenagel condensation is regarded as a nucleophilic addition of a C–H acidic compound to a carbonyl group of an aldehyde or ketone to form a double bond under elimination of one equivalent of water (Scheme 1) [5,8,9].

In recent years a wide scope of different substrates as well as different bases and reaction conditions has been reported. Broadly applied is the use of piperidine as a base. In some cases the reaction is carried out at room temperature [10], and in other cases the use of acetic acid as an additive next to a nonpolar solvent like benzene or toluene under reflux conditions was established [11,12,13,14]. Green alternatives to classical Knoevenagel condensation avoid the use of organic solvents, use catalysts from naturally occurring compounds, improve the atomic economy of the reaction or are carried out at mild reaction conditions that thus reduce energy consumption [15,16,17,18].

In general, reductive condensations are thoroughly known. However, most of these transformations are two-step reactions, where the reduction, with an external reducing agent, takes place after the condensation. Methods using inorganic reducing agents in combination with the Knoevenagel condensation, like zinc in acetic acid [19] and sodium borohydride [20], as well as enzymatic reductions [21] and hydrogenations with palladium were published [22]. In addition, one-pot synthetic routes to reduced compounds were investigated as well [23]. 

Phosphine Lewis bases that catalyze internal redox transformations are known in literature [24]. However, only two examples of phosphine-promoted reductive aldol reactions have been published [25]. Recently a homogenous phosphine-promoted reductive aldol reaction of activated *α,β*-unsaturated carbonyls with aldehydes was reported by Gu et al. An external triphenylphosphine molecule was applied to perform a phospha-Michael addition leading to a zwitterionic enolate, followed by P–O bond formation and C–P bond cleavage to give reduced aldol compounds [26]. A similar mechanism was investigated by Satpathi et al., containing an intermolecular 1,6-addition of a phosphine, later on eliminated as a phosphine oxide [27].

Herein we report that this kind of mechanism can also be found in an intramolecular phosphine-promoted Knoevenagel redox reaction, where the phosphine oxide moiety remains in the molecule, in contrast to the so far reported systems. There are a few further examples of internal redox reactions which have been catalyzed by, for example, carbenes or metal complexes [28,29,30,31]. However, the herein-described type of internal redox reaction, promoted by a triphenylphosphine moiety, is unprecedented and will help for planning new synthetic routes to new phosphine ligands.

## 2. Results and Discussion

**Scheme 2:**
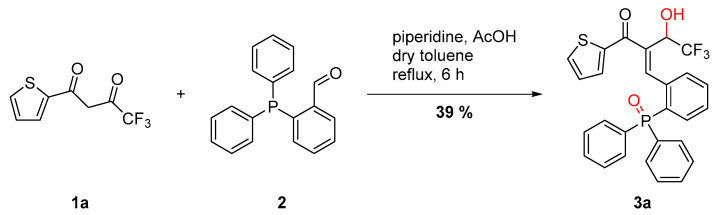
Knoevenagel reaction of TTA with 2-(diphenylphosphaneyl)benzaldehyde.

During our research in the field of a Knoevenagel reaction with 4,4,4-trifluoro-1-(thiophen-2-yl)butane-1,3-dione (TTA) and 2-(diphenylphosphaneyl)benzaldehyde in the presence of piperidine and acetic acid in order to prepare new phosphine ligands, we discovered the reduction of the keto function in the α-position to the CF_3_ group of TTA, whereas the phosphine atom of the triphenylphosphine moiety was transformed to the phosphine oxide with a yield of 39% (Scheme 2).

The reaction was also carried out under Dean-Stark conditions with dry toluene. Even then, the product **3a** was formed in identical yield, indicating that the water in situ produced by the Knoevenagel condensation participates rapidly in the reaction. In order to investigate this unusual type of reaction in detail, different ketones, acids, bases and reaction conditions were explored in the transformation and the resulting products were analyzed with ^1^H-, ^13^C and ^31^P-NMR spectroscopy (see Supplementary Materials). In addition, the reaction was carried out under dry and deoxygenated conditions and was followed by in situ ^31^P-NMR. The spectra indicate the formation of the phosphine oxide **3a** without oxygen from the atmosphere, which leads to the assumption that water formed in the condensation reaction is the oxygen source for the oxidation of the phosphine.

### 2.1. Scope of Diketones

**Scheme 3:**
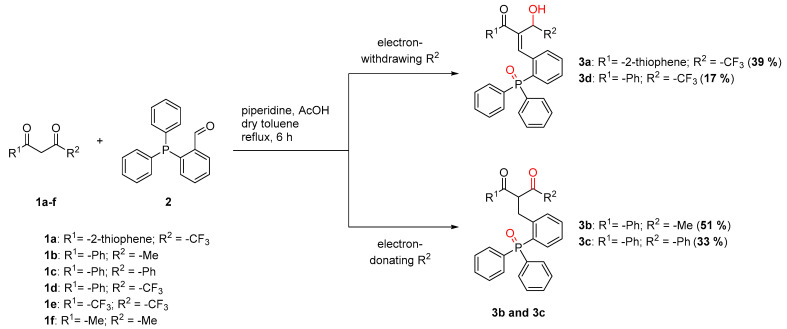
Evaluation of different ketones in the Knoevenagel reaction.

In addition to TTA other diketones bearing different functional groups, either electron-withdrawing (-CF_3_) or electron-donating in nature (-Ph or -Me), were evaluated (Scheme 3).

In the case that the reaction was performed with a diketone bearing a CF_3_ group, it was observed that the keto function in α-position to the CF_3_ group is reduced, which is in agreement with the experiment using TTA as the diketone (products **3a** and **3d**). The structures obtained with electron-donating groups attached to the diketone moiety still contain both oxo-groups. However, the double bond which was introduced by the Knoevenagel reaction was not found and instead was reduced to the corresponding single bond (products **3b** and **3c**). If the diketones **1e** and **1f** were used, no product could be obtained, even after an increased reaction time of 48 h.

### 2.2. Reaction with Benzaldehyde

**Scheme 4:**
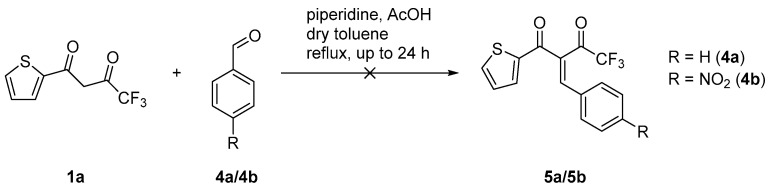
Reaction of TTA (**1a**) with aldehydes without a phosphine moiety (**4a**/**4b**).

To determine the importance of the phosphine group in the aldehyde component, further reactions with benzaldehydes were carried out. The reaction of TTA with benzaldehyde (**4a**) and 4-nitrobenzaldehyde (**4b**) did not give any product under the established reaction conditions (Scheme 4). Besides the unreacted starting material, only an increasing decomposition of **1a** was observed. To the best of our knowledge the preparation of diketones **5a** and **5b** is not presented in literature so far.

**Scheme 5:**
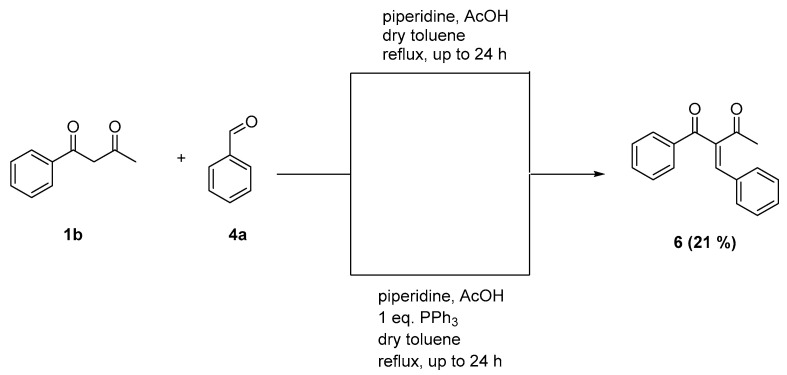
Reaction of benzoylacetone (**1b**) with benzaldehyde (**4a**).

The reaction of benzoylacetone with benzaldehyde resulted in the formation of the originally expected Knoevenagel product with a yield of 21% (Scheme 5).

Furthermore, the reaction was carried out with one additional equivalent of triphenylphosphine, to investigate if the use of an external phosphine source leads to a redox reaction as well. Again, diketone **6** was isolated, which indicates that the reaction pathway of the reductive Knoevenagel reaction needs an intramolecular phosphine moiety in the aldehyde in order to reduce either the keto function or the double bond of the molecule.

### 2.3. Scope of Acids

**Scheme 6:**
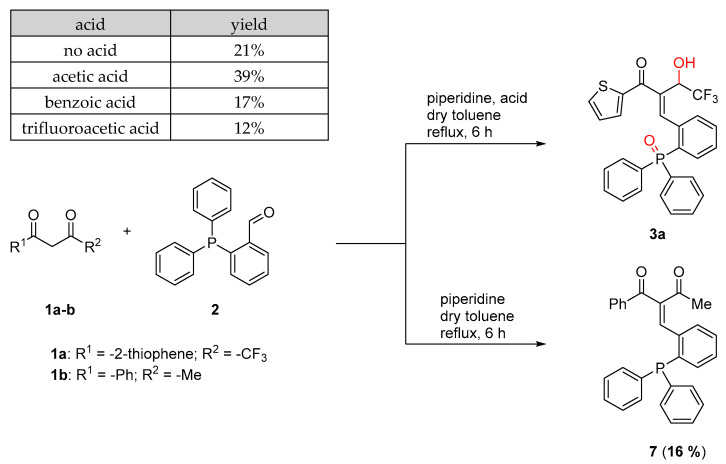
Scope of acids in the reductive Knoevenagel reaction.

In addition, different acids were investigated in the reaction instead of acetic acid, to evaluate their influence on the overall performance of the transformation (Scheme 6).

The yield decreased in all reactions when compared to those where acetic acid was applied as an acid. In all those reactions, unreacted starting material as well as decomposition products were observed. In the reaction of TTA with aldehyde **2**, the choice of the acid had no influence on the product formation. Product **3a** was obtained in almost all cases. Interestingly, no oxidation of the phosphine and therefore no reduction of the keto function or double bond was observed, if the reaction was carried out with benzoylacetone without the use of any acid and increased reaction time to 16 h (product **7**).

### 2.4. Scope of Bases

Next, a variety of different bases was evaluated in the established reaction of TTA with aldehyde **2**. Neither bases with an amine functionality like triethylamine, DABCO or pyrrolidine nor bases with a pyridine moiety, like pyridine itself or lutidine led to any product formation. In addition, cesium fluoride was used as an inorganic base. Similar to the use of the other bases, no product was obtained. Without any base the reaction did not proceed either. In all cases, only the decomposition of the starting material was observed and unreacted starting material was recovered.

### 2.5. Proposed Mechanism

**Scheme 7:**
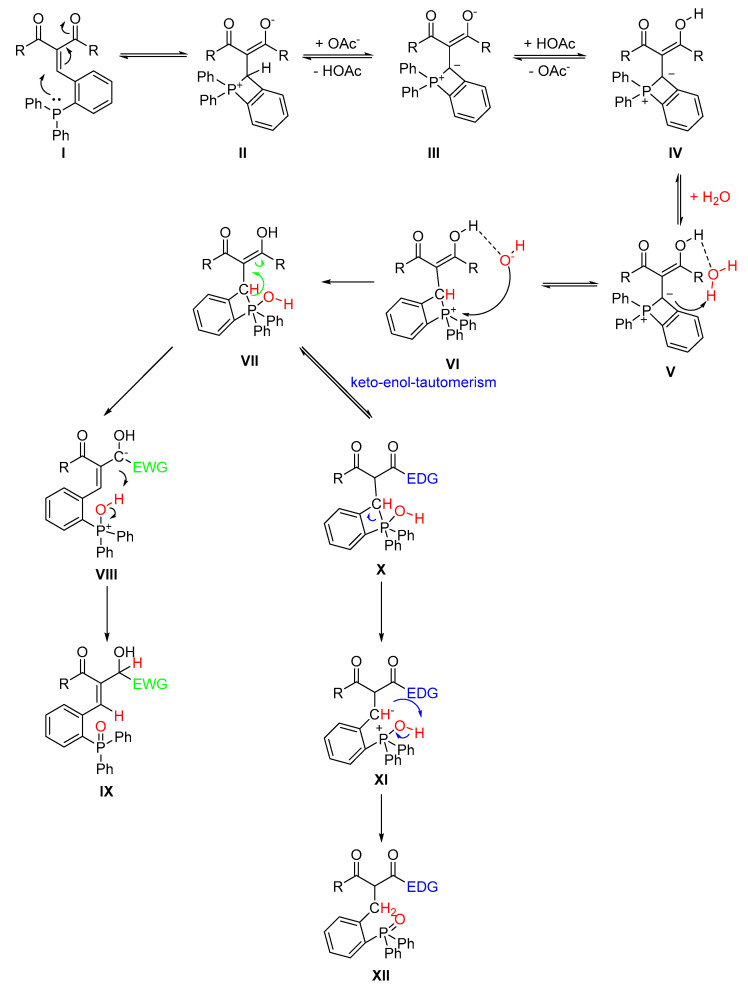
Proposed Mechanism.

Considering the experimental results, the following mechanism (Scheme 7) can be postulated. The first step of the reaction is the regular formation of the Knoevenagel product (**I**). In the next step a four-membered ring is formed due to the attack of the electron pair of the phosphorous atom at the double bond. This type of addition is similar to the first step of a phosphine-mediated Morita-Baylis-Hillman-type reaction, starting with the attack of a phosphine to an α,β-unsaturated carbonyl group [26]. This way the zwitterion(**II**) is formed. The formation of a six-membered ring formed by the attack of the phosphine at the carbonyl group is also conceivable, but the formation of the four-membered ring is entropically preferred and is, as a short lived intermediate, highly conceivable, even though the ring is highly strained. In addition, a phosphine atom would attack more favorably the softer unsaturated β-position rather than a carbonyl atom. Furthermore, a six-membered ring of this type is known to be highly water sensitive [32]. The four membered ring is deprotonated by an acetate ion (**III**). For **III**, an ylid structure is shown, yet an ylene structure is also possible since an isolated analogue of **III** has been reported in the literature [33]. The formed acetic acid protonates the alcoholate function (**IV**). The carbanion is protonated by a proton of a water molecule, formed by the Knoevenagel reaction (**V**). The remaining hydroxy group attacks the positively charged phosphorous atom (**VI**). Different products are formed from intermediate **VII** depending on the electronic nature of the substituent. Electron-withdrawing groups lead to the observed redox product whereas electron-donating groups promoted the formation of one of the keto-enol-tautomers in larger amount. In detail, if the substituent is an electron-withdrawing group, there is a negative charge formed at the former carbonyl carbon atom after the opening of the four-membered ring, which is stabilized by the substituent (**VIII**). The negative charge cleaves of the proton from the oxygen atom, which is bound to the phosphorous atom, to form the product with a reduced ketone function and phosphine oxide moiety (**IX**).

If the substituent is an electron-donating group, the ketone is formed by tautomerism (**X**). In this case, the negative charge is located at the carbon atom of the former double bond, after the four-membered ring was opened (**XI**). Cleavage of the proton by the carbanion leads to the product with single bond and the phosphine oxide functionality (**XII**).

## 3. Materials and Methods

Commercially available compounds were used without further purification. All solvents used were dried using the MP5 solvent purification system from INERT TECHNOLOGY over special aluminum oxide columns and under a nitrogen atmosphere. All reaction mixtures were degassed prior to the reaction. The ^1^H, ^13^C and ^31^P NMR spectra were recorded with the FT-NMR spectrometer “BRUKER AVANCE” with 400 MHz proton frequency and the “BRUKER AVANCE III” with a 600 MHz proton frequency. The chemical shifts are expressed in ppm (*δ*-scale). Assignments are based on HSQC and HMBC spectra. The HRMS spectra were recorded with the “Impact II” from BRUKER. The recording was done as ESI mass spectra. The melting points were measured with the DSC6 device from PERKIN ELMER and determined via the onset temperatures.

### 3.1. General Procedure for the Knoevenagel Reaction

Unless otherwise stated, reactions were performed as follows. 1 eq of the acid and 1 eq of the base were added to an ice-cold solution of the corresponding aldehyde (1 eq) and the diketone (1 eq) in dry toluene under a nitrogen atmosphere (sometimes non-dry toluene was used with the Dean-stark set-up). The mixture was allowed to heat up to room temperature and heated to reflux for 6 h afterwards. The mixture was cooled to room temperature and diluted with water, neutralized with hydrochloric acid and extracted with dichloromethane. The combined organics were dried over sodium sulfate and the crude product was purified by flash column chromatography.

### 3.2. 2-(2-(Diphenylphosphoryl)benzylidene)-4,4,4-trifluoro-3-hydroxy-1-(thiophen-2-yl)butan-1-one (***3a***)

From **1a** (5.0009 g, 17.22 mmol) and **2** (3.8302 g, 17.22 mmol), purified by silica gel column chromatography using petroleum ether/ethyl acetate 2:1 as eluent to obtain 3.1723 g (39%) of compound **3a** as a pale-yellow solid; m.p. 158 °C. ^1^H-NMR (600 MHz, CDCl_3_): *δ* = 7.99 (m, 1H, ArH), 7.85 (s, 1H, CH), 7.72–7.39 (m, 14H, ArH), 7.19 (m, 1H, ArH), 7.15 (m, 1H, ArH), 4.84 (m, 1H, CH) ppm. ^13^C-NMR (150 MHz, CDCl_3_): *δ* = 190.3, 142.6, 142.0, 138.3, 137.3, 135.9, 133.7, 133.5, 132.5-132.3 (m, ArC), 132.1–131.7 (m, ArC), 131.4 (d, ^2^*J*_C-P_ = 11.8 Hz), 131.2, 130.8, 130.5 (d, ^3^*J*_C-P_ = 9.3 Hz), 128.9–128.8 (m, ArC), 128.6–128.5 (m, ArC), 128.4, 70.0 (q, *J*_C-F_ = 32.6 Hz, CF_3_) ppm. ^31^P-NMR (243 MHz, CDCl_3_): *δ* = 31.2 ppm. HRMS (ESI): *m/z* calcd for C_27_H_20_F_3_NaO_3_PS [M + Na]^+^ 535.0715, found 535.0731.

### 3.3. 2-(2-(Diphenylphosphoryl)benzyl)-1-phenylbutane-1,3-dione (***3b***)

From **1b** (165.6 mg, 1.02 mmol) and **2** (293.4 mg, 1.01 mmol), reaction time: 16 h, purified by silica gel column chromatography using petroleum ether/ethyl acetate 1:1 as eluent to obtain 232.9 mg (51%) of compound **3b** as a pale yellow solid; m.p. 283 °C. ^1^H-NMR (600 MHz, CDCl_3_): *δ* = 7.88 (dd, *J_H,H_* = 8.0, 1.3 Hz, 2H, ArH), 7.80–7.22 (m, 15H, ArH), 7.08–7.06 (m, 1H, ArH), 7.01–6.89 (m, 1H, ArH), 5.74 (dd, *J_H,H_* = 9.3, 5.2 Hz, 1H, CH), 3.48 (dd, *J_H,H_* = 13.6, 5.2 Hz, 1H, CH_2_), 3.36 (dd, *J_H,H_* = 13.6, 9.3 Hz, 1H, CH_2_), 2.17 (s, 3H, CH_3_) ppm. ^13^C-NMR (150 MHz, CDCl_3_): *δ* = 203.6, 196.6, 143.8, 137.1, 133.8, 133.4 (d, *J_C,P_* = 10.4 Hz, ArC), 133.2, 133.1 (d, *J_C,P_* = 18.4 Hz, ArC), 132.8, 132.1–131.9 (m), 131.7 (d, *J_C,P_* = 10.0 Hz, ArC), 131.2 (d, *J_C,P_* = 100.0 Hz, ArC), 128.8, 128.7–128.5 (m), 128.3 (d, *J_C,P_* = 16.2 Hz, ArC), 126.1 (d, *J_C,P_* = 13.0 Hz, ArC), 62.6, 34.0 (d, *J_C,P_* = 4.4 Hz), 29.9 ppm. ^31^P-NMR (243 MHz, CDCl_3_): *δ* = 32.1 ppm. HRMS (ESI): *m/z* calcd for C_29_H_25_NaO_5_P [M + Na]^+^ 475.1434, found 475.1447.

### 3.4. 2-(2-(Diphenylphosphoryl)benzyl)-1,3-diphenylpropane-1,3-dione (***3c***)

From **1c** (394.1 mg, 1.76 mmol) and **2** (517.4 mg, 1.78 mmol), reaction time: 16 h, purified by silica gel column chromatography using petroleum ether/ethyl acetate 5:1 as eluent to obtain 297.3 mg (33%) of compound **3c** as a pale yellow solid; m.p. 171 °C. ^1^H-NMR (600 MHz, CDCl_3_): *δ* = 8.18–7.86 (m, 4H, ArH), 7.63 (ddt, *J_H,H_* = 12.0, 6.9, 1.4 Hz, 4H, ArH), 7.59–7.51 (m, 2H, ArH), 7.49–7.36 (m, 6H, ArH), 7.37–7.28 (m, 4H, ArH), 7.26–7.23 (m, 1H, ArH), 7.17 (tt, *J_H,H_* = 7.6, 1.6 Hz, 1H, ArH), 6.98 (tdd, *J_H,H_* = 7.5, 2.6, 1.3 Hz, 1H, ArH), 6.93 (ddd, *J_H,H_* = 14.2, 7.7, 1.5 Hz, 1H, ArH), 6.82 (t, *J_H,H_* = 7.8 Hz, 1H, CH), 3.49 (d, *J_H,H_* = 7.8 Hz, 2H, CH_2_) ppm. ^13^C-NMR (150 MHz, CDCl_3_): *δ* = 196.6, 143.2 (d, *J*_C,P_ = 8.0 Hz, ArC), 136.7, 133.9 (d, *J*_C,P_ = 10.0 Hz, ArC), 133.7 (d, *J*_C,P_ = 13.2 Hz, ArC), 133.0, 132.5 (d, *J*_C,P_ = 103.8 Hz, ArC), 132.0 (d, *J*_C,P_ = 2.4 Hz, ArC), 131.9 (d, *J*_C,P_ = 3.1 Hz, ArC), 131.8 (d, *J*_C,P_ = 3.0 Hz, ArC), 131.3 (d, *J*_C,P_ = 100.9 Hz, ArC), 128.9, 128.7 (d, *J*_C,P_ = 12.6 Hz, ArC), 128.4, 56.8, 36.0 (d, *J*_C,P_ = 4.8 Hz, CH_2_) ppm. ^31^P-NMR (243 MHz, CDCl_3_): *δ* = 32.3 ppm. HRMS (ESI): *m/z* calcd for C_34_H_27_NaO_3_P [M + Na]^+^ 537.1590, found 537.1591.

### 3.5. 2-(2-(Diphenylphosphoryl)benzylidene)-4,4,4-trifluoro-3-hydroxy-1-phenylbutan-1-one (***3d***)

From **1d** (401.5 mg, 1.86 mmol) and **2** (542.1 mg, 1.87 mmol) and, reaction time: 16 h, purified by silica gel column chromatography using petroleum ether/ethyl acetate 5:1 as eluent to obtain 156.2 mg (17%) of compound **3d** as a pale yellow solid; m.p. 269 °C. ^1^H-NMR (600 MHz, CDCl_3_): *δ* = 7.85–7.70 (m, 2H, ArH), 7.69–7.60 (m, 4H, ArH), 7.60–7.53 (m, 4H, ArH), 7.53–7.46 (m, 4H, ArH), 7.46–7.40 (m, 4H, ArH), 7.40–7.35 (m, 1H, ArH), 7.17 (ddd, *J_H,H_* = 13.9, 7.7, 1.3 Hz, 1H, ArH), 4.96 (q, *J_H,F_* = 7.2 Hz, 1H, CH) ppm. ^13^C-NMR (150 MHz, CDCl_3_): *δ* = 198.9, 144.1 (d, *J*_C,P_ = 4.4 Hz, CH), 138.4 (d, *J*_C,P_ = 6.7 Hz, ArC), 135.9, 134.1, 133.7 (d, *J*_C,P_ = 11.6 Hz, ArC), 133.4, 132.4 (d, *J*_C,P_ = 2.6 Hz, ArC), 132.3 (d, *J*_C,P_ = 3.1 Hz, ArC), 131.9, 131.8, 131.7 (d, *J*_C,P_ = 9.9 Hz, ArC), 131.4 (d, *J*_C,P_ = 31.0 Hz, ArC), 131.0 (d, *J*_C,P_ = 48.0 Hz., ArC), 130.3, 130.2, 128.8 (d, *J*_C,P_ = 12.1 Hz, ArC), 128.5, 124.5 (q, *J*_C,F_ = 282.4 Hz, CF_3_), 69.8 (q, *J*_C,F_ = 32.3 Hz, CH) ppm. ^31^P-NMR (243 MHz, CDCl_3_): *δ* = 31.0 ppm. HRMS (ESI): *m/z* calcd for C_29_H_22_F_3_NaO_3_P [M + Na]^+^ 529.1151, found 529.1164.

### 3.6. 2-Benzylidene-1-phenylbutane-1,3-dione (***6***)

From **1b** (288.5 mg, 1.78 mmol) and **4a** (180 μL, 1.78 mmol) and 1 equiv. PPh_3_, reaction time: 16 h, purified by silica gel column chromatography using petroleum ether/ethyl acetate 15:1 as eluent to obtain 92.5 mg (21%) of compound **6** as a yellow solid; ^1^H-NMR (400 MHz, CDCl_3_): *δ* = 7.98–7.77 (m, 2H, ArH), 7.72 (s, 1H, CH), 7.55–7.42 (m, 1H, ArH), 7.37–7.30 (m, 2H, ArH), 7.29–7.24 (m, 2H, ArH), 7.22–7.12 (m, 3H, ArH), 2.32 (s, 3H, CH_3_) ppm. ^13^C-NMR (100 MHz, CDCl_3_): *δ* = 198.1, 195.9, 141.3, 139.6, 136.0, 134.1, 132.9, 130.6, 130.4, 129.2, 129.0, 128.9, 27.3 ppm. Spectroscopic data are in agreement with the literature [17].

### 3.7. 2-(2-(Diphenylphosphaneyl)benzylidene)-1-phenylbutane-1,3-dione (***7***)

From **1b** (142.9 mg, 0.88 mmol) and **2** (256.1 mg, 0.88 mmol), without the use of an acid, reaction time: 24 h purified by silica gel column chromatography using petroleum ether/ethyl acetate 2:1 as eluent to obtain 63.9 mg (16%) of compound **7** as a pale yellow solid; m.p. 283 °C. ^1^H-NMR (600 MHz, CDCl_3_): *δ* = 8.57 (d, *J_H,H_* = 5.2 Hz, 1H, CH), 7.57–7.48 (m, 2H, ArH), 7.46–7.35 (m, 8H, ArH), 7.30 (td, *J_H,H_* = 8.0, 1.5 Hz, 4H, ArH), 7.23–7.17 (m, 2H, ArH), 7.17–7.07 (m, 2H, ArH), 6.96–6.88 (m, 1H, ArH), 2.37 (s, 3H, CH_3_) ppm. ^13^C-NMR (150 MHz, CDCl_3_): *δ* = 197.3, 196.7, 140.4 (d, *J*_C,P_ = 2.0 Hz), 140.0 (d, *J*_C,P_ = 27.4 Hz, CH), 138.7 (d, *J*_C,P_ = 14.6 Hz, ArC), 137.5 (d, *J*_C,P_ = 22.1 Hz, ArC), 136.0, 135.3 (d, *J*_C,P_ = 8.9 Hz, ArC), 134.3 (d, *J*_C,P_ = 19.5 Hz, ArC), 133.6, 133.3, 130.0, 129.6 (d, *J*_C,P_ = 3.9 Hz, ArC), 129.3, 129.0 (d, *J*_C,P_ = 4.2 Hz, ArC), 128.9 (d, *J*_C,P_ = 7.3 Hz, ArC), 128.6, 26.8 ppm. ^31^P-NMR (243 MHz, CDCl_3_): *δ* = -14.8 ppm. HRMS (ESI): *m/z* calcd for C_29_H_23_NaO_4_P [M + Na]^+^ 457.1328, found 457.1314.

## 4. Conclusions

In conclusion, we reported an intramolecular Knoevenagel redox reaction, which is promoted by an intramolecular oxidation of a phosphine source, gained after a conventional Knoevenagel product formation. The new products were formed in up to 51% yield. In order to gain insights into the mechanism on this reaction we performed different reactions with a variety of diketones, aldehydes, acids and bases and analyzed the products via ^1^H-, ^13^C- and ^31^P-NMR spectroscopy. The results indicate that the electronic structure of the diketone has a significant influence on the structure of the product. The use of an electron-withdrawing group leads to a reduction of the keto group, whereas electron-donating substituents of the diketone lead to the reduction of the double bond formed by the Knoevenagel reaction. While the reduction of one of the aforementioned functions occurred, the phosphine is oxidized. It is assumed that water formed during the Knoevenagel reaction is the source for both reduction and oxidation. In addition to the direct synthesis of the reported new products in medium yield, the presented findings are important for planning new synthetic routes to phosphine-containing compounds such as new ligands for metalorganic catalysts.

## Data Availability

Not applicable.

## Supplementary Materials

The following supporting information can be downloaded at: https://www.mdpi.com/article/10.3390/molecules27154875/s1. ^1^H, ^13^C, ^31^P NMR and ESI-HRMS Spectra.

## Sample Availability

Samples of the compounds are available from the authors.
