# Supplementary material for: Intramolecular Phosphine-Promoted Knoevenagel Based Redox-Reaction
Source: Molecules. 2022 Jul 29;27(15):4875. doi: 10.3390/molecules27154875 (PMC9370043; doi:10.3390/molecules27154875)
Supplement: Supplementary file 1 [file molecules-27-04875-s001.zip › molecules-1787647-supplementary.pdf]

Supporting Information

Intramolecular Phosphine-promoted Knoevenagel based Redox-Reaction

N. Feuge[1], J. C. Namyslo[1], D. E. Kaufmann[1], R. Wilhelm*[1]

Address: [1]Institute of Organic Chemistry, Clausthal University of Technology, Leibnizstrasse 6, D-38678 Clausthal-Zellerfeld, Germany

Email: rene.wilhelm@tu-clausthal.de

* Corresponding author

Content

^{1}H, ^{13}C, ^{31}P NMR and ESI-HRMS Spectra **S2-S13**

Spectra of 3a:

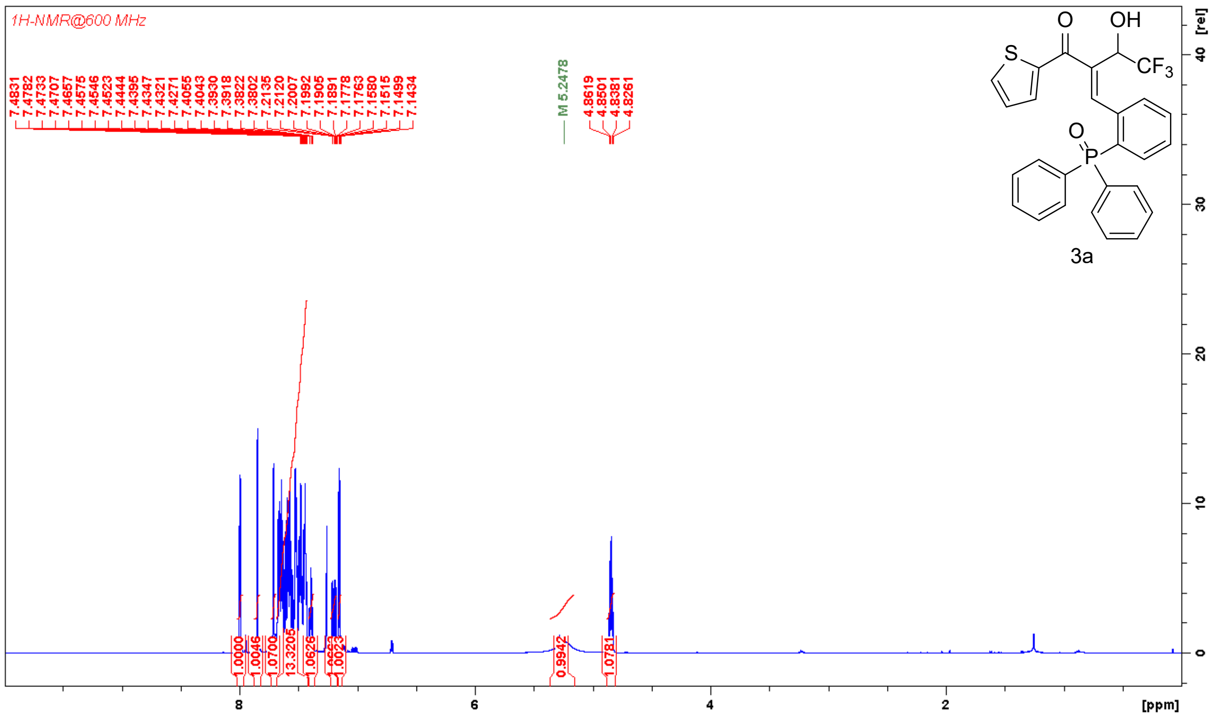

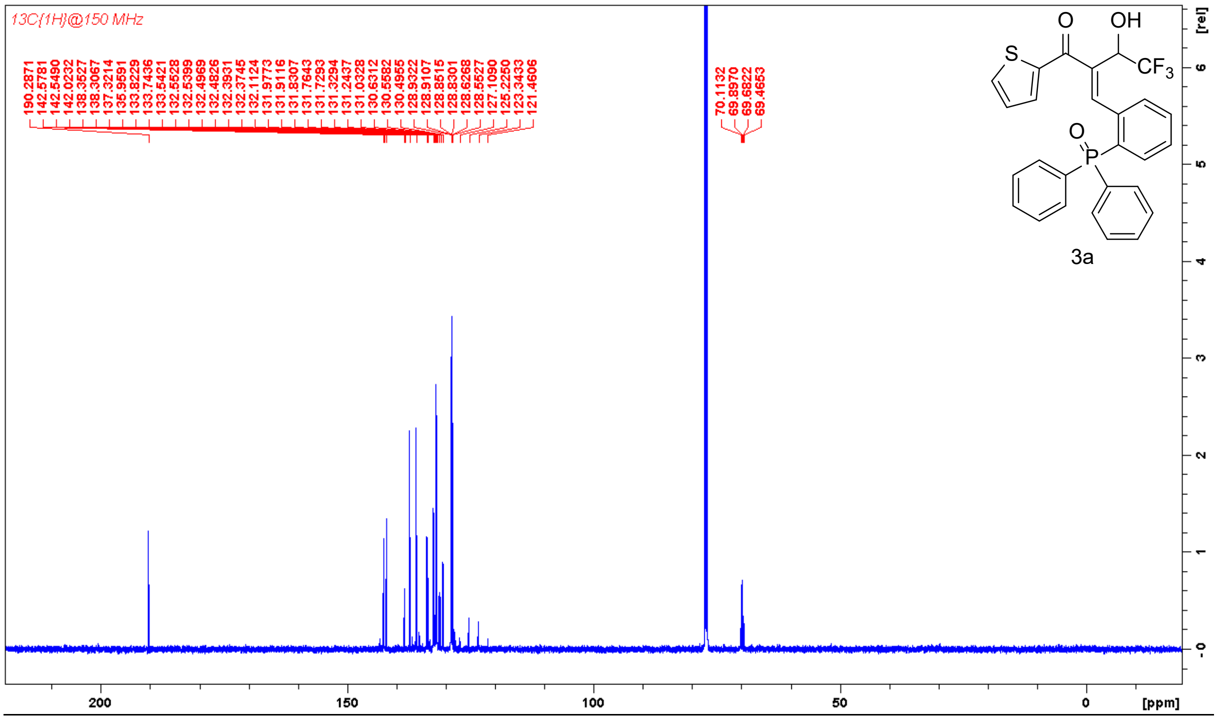

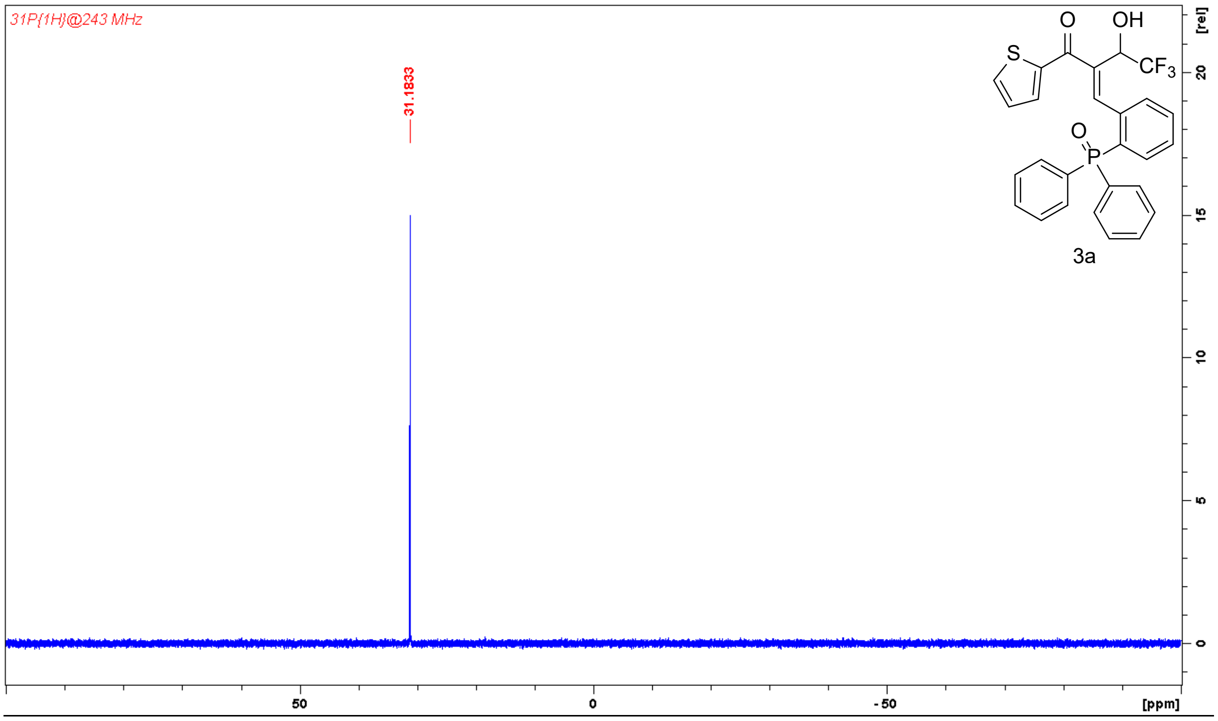
31P{1H}@243 MHz
31.1833
[rel]
20
15
10
5
0
50
0
- 50
[ppm]
O
OH
S
CF3
O
P
3a

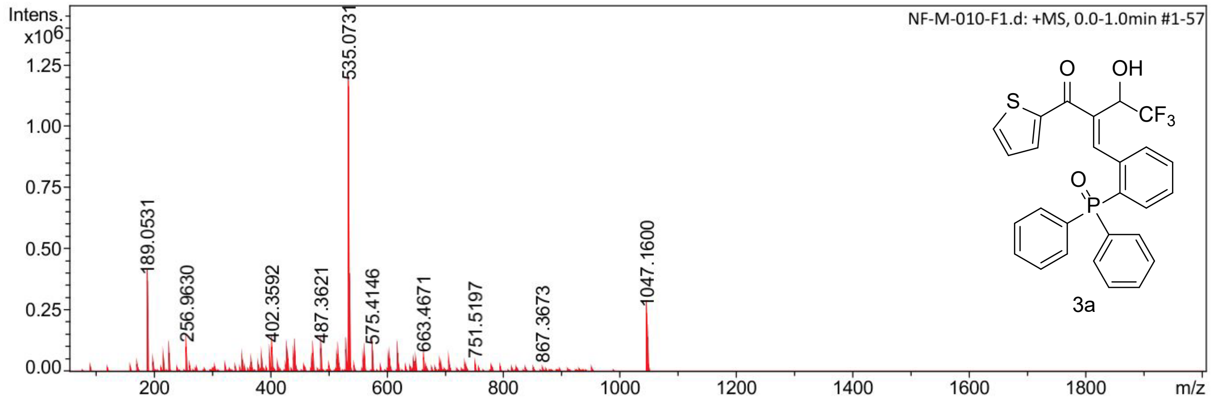
Intens.
x10^6
1.25
1.00
0.75
0.50
0.25
0.00
NF-M-010-F1.d: +MS, 0.0-1.0min #1-57
535.0731
189.0531
256.9630
402.3592
487.3621
575.4146
663.4671
751.5197
867.3673
1047.1600
200
400
600
800
1000
1200
1400
1600
1800
m/z
O
OH
S
CF3
O
P
3a

Spectra of 3b:

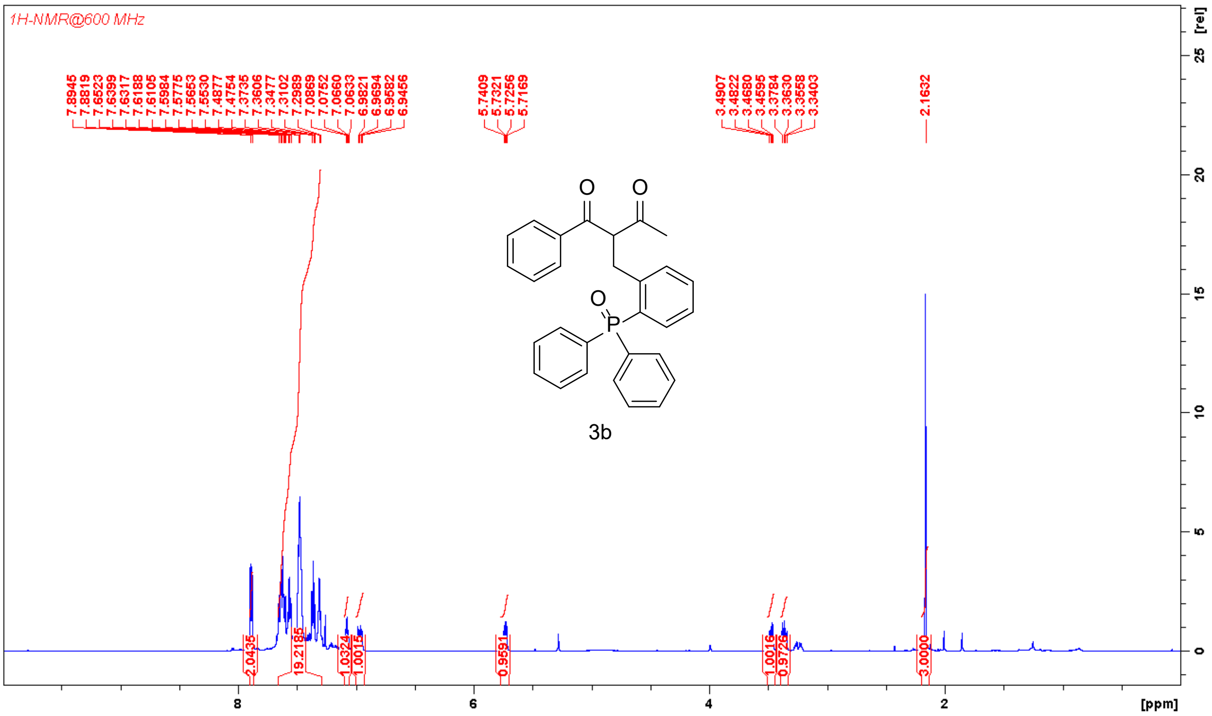

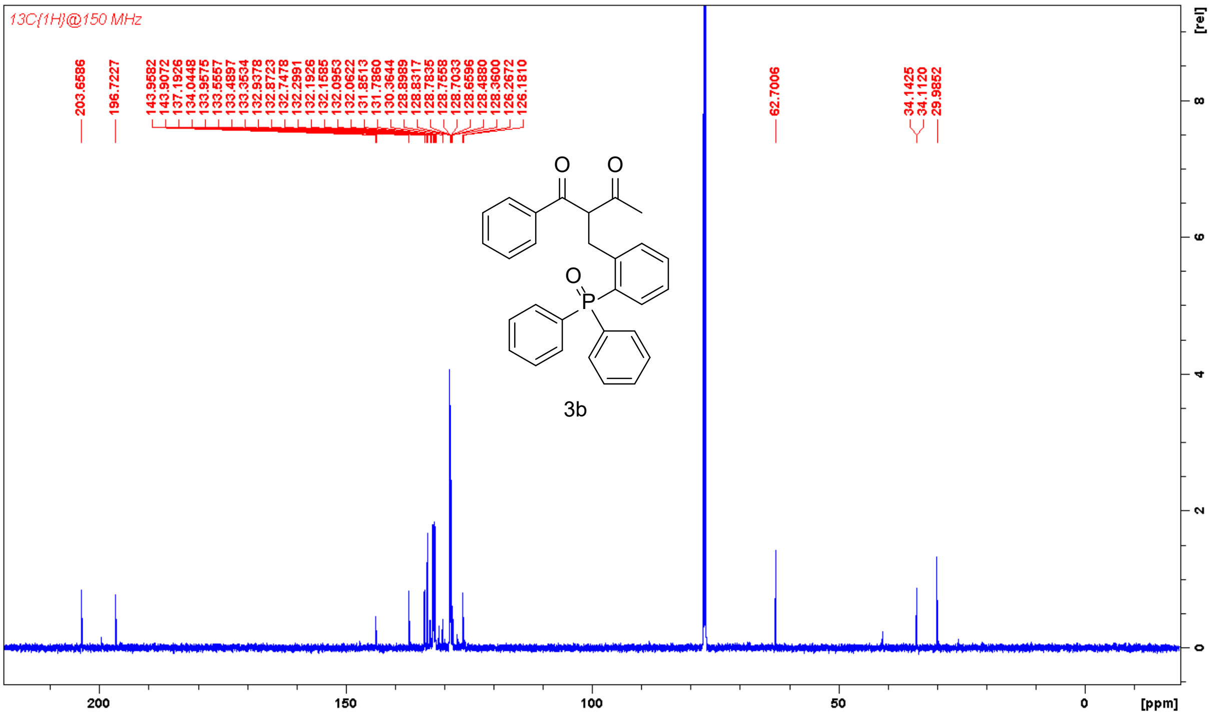

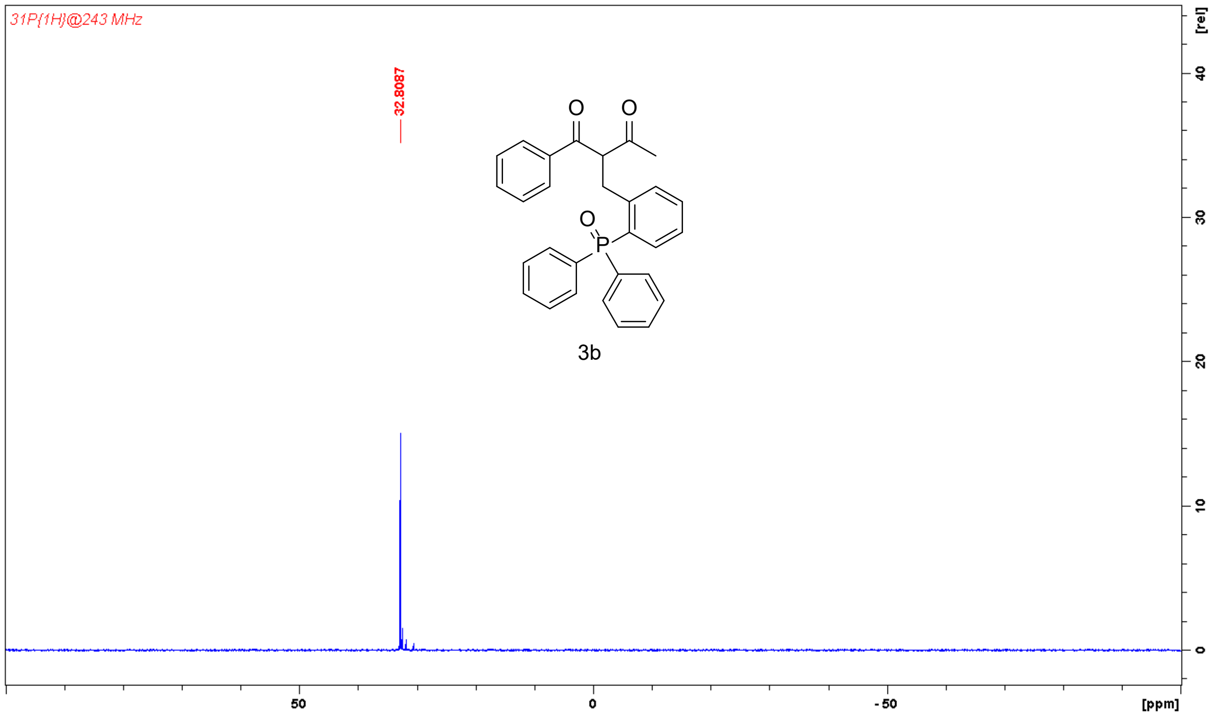
31P{1H}@243 MHz
32.8087
3b
[rel]
40
30
20
10
0
50
0
- 50
[ppm]

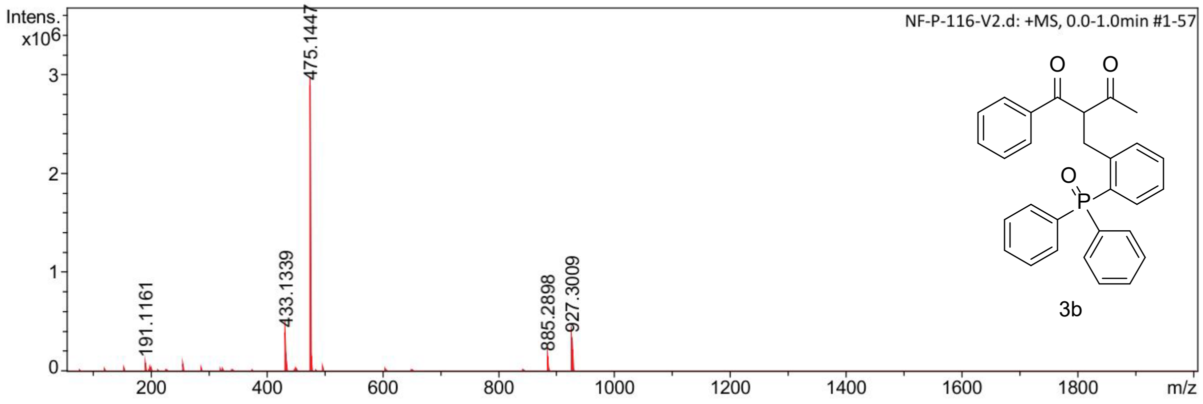
Intens.
x10^6
3
2
1
0
NF-P-116-V2.d: +MS, 0.0-1.0min #1-57
475.1447
433.1339
191.1161
885.2898
927.3009
3b
200
400
600
800
1000
1200
1400
1600
1800
m/z

Spectra of 3c:

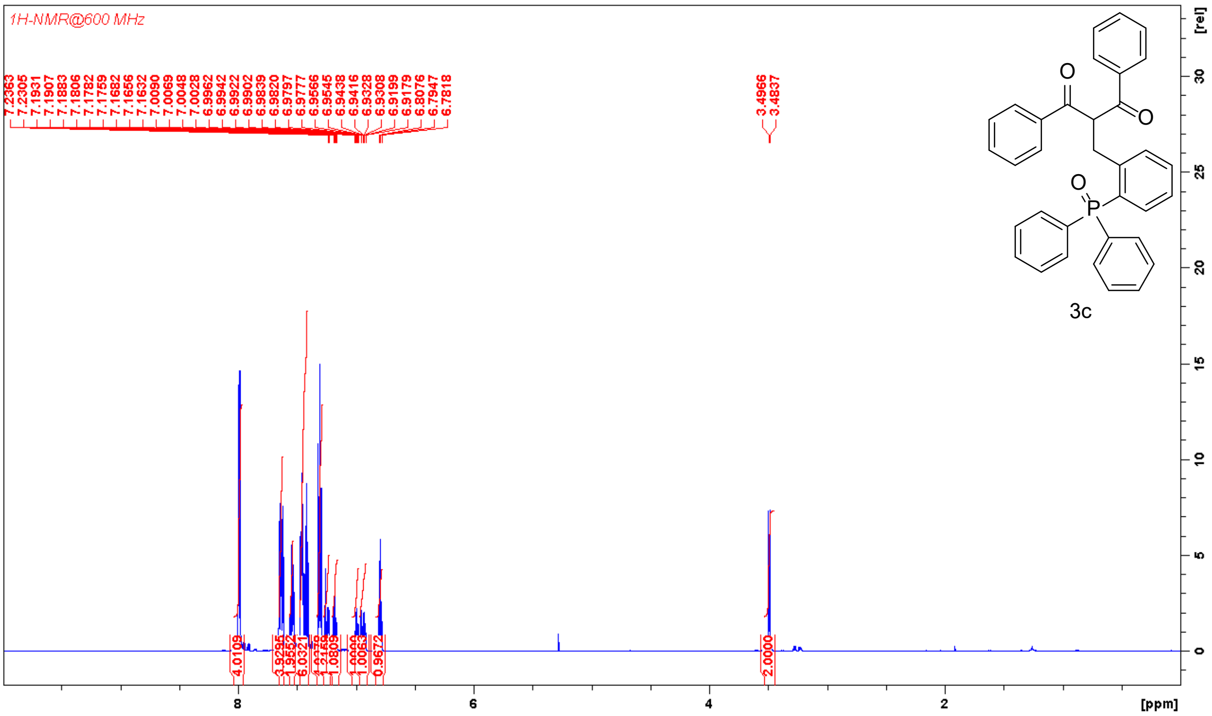

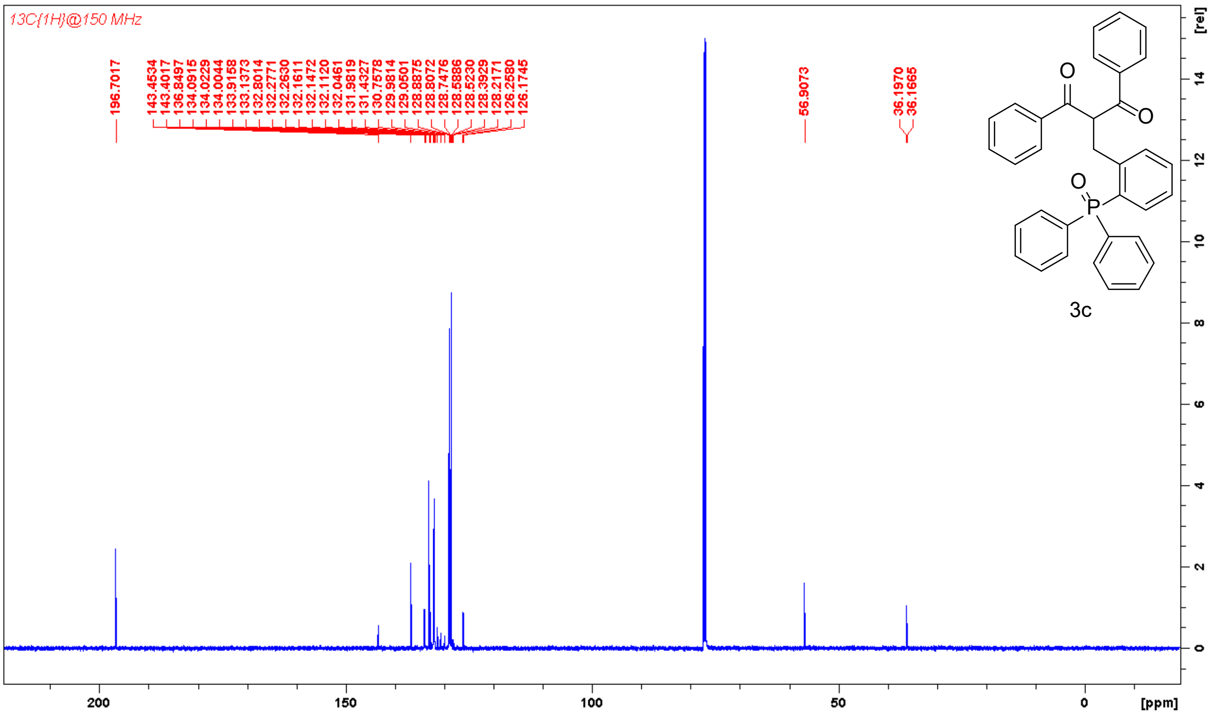

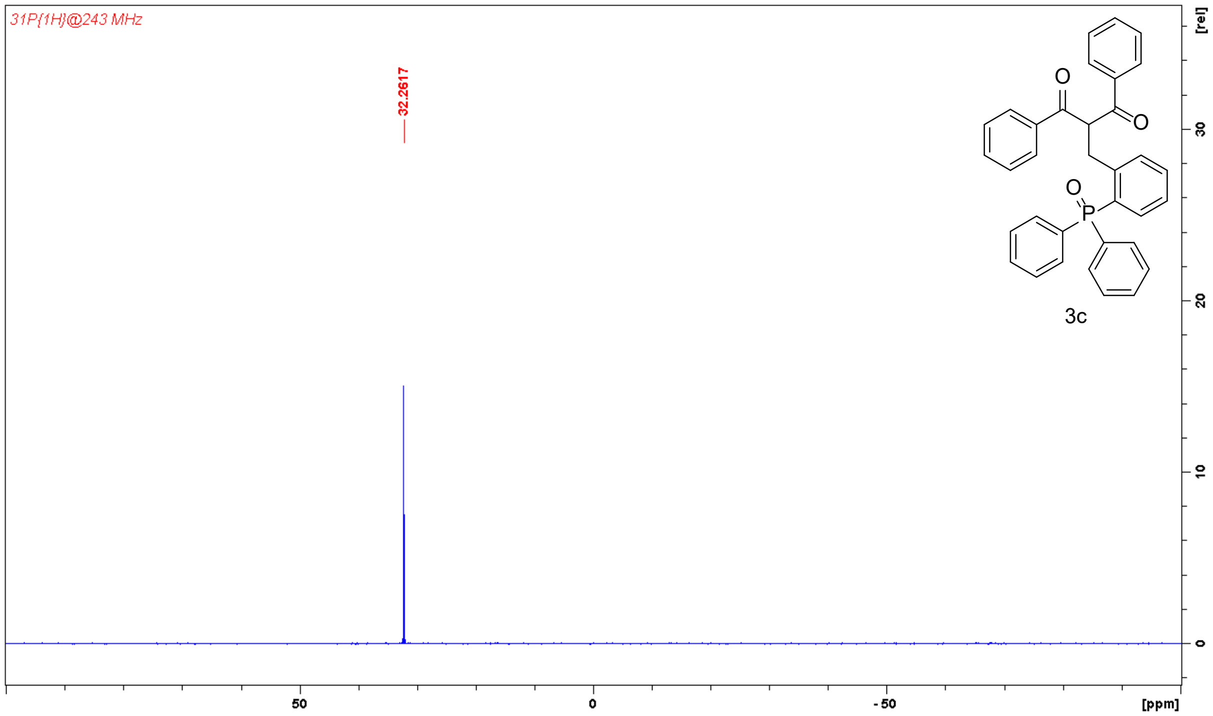
31P{1H}@243 MHz
32.2617
[rel]
30
20
10
0
50
0
- 50
[ppm]
O
O
O
P
3c

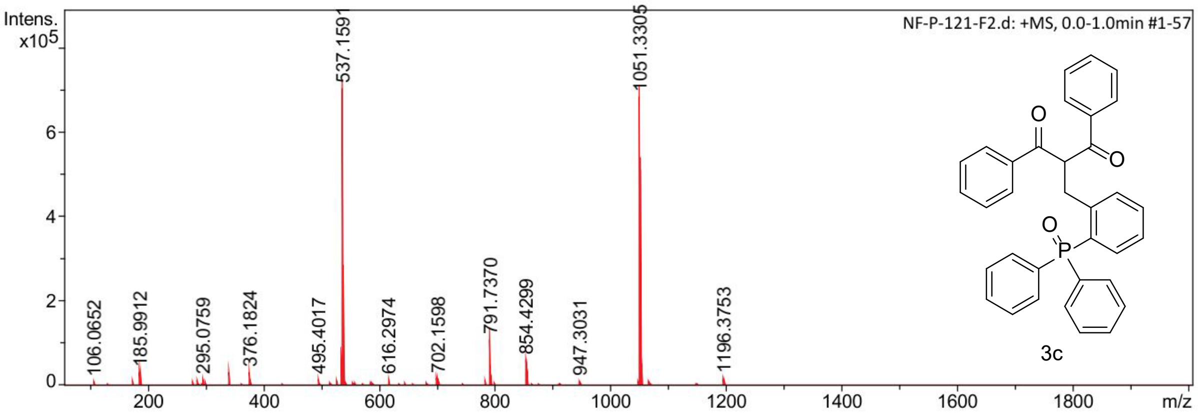
Intens.
x10^5
6
4
2
0
537.1591
1051.3305
NF-P-121-F2.d: +MS, 0.0-1.0min #1-57
106.0652
185.9912
295.0759
376.1824
495.4017
616.2974
702.1598
791.7370
854.4299
947.3031
1196.3753
200
400
600
800
1000
1200
1400
1600
1800
m/z
O
O
O
P
3c

Spectra of 3d:

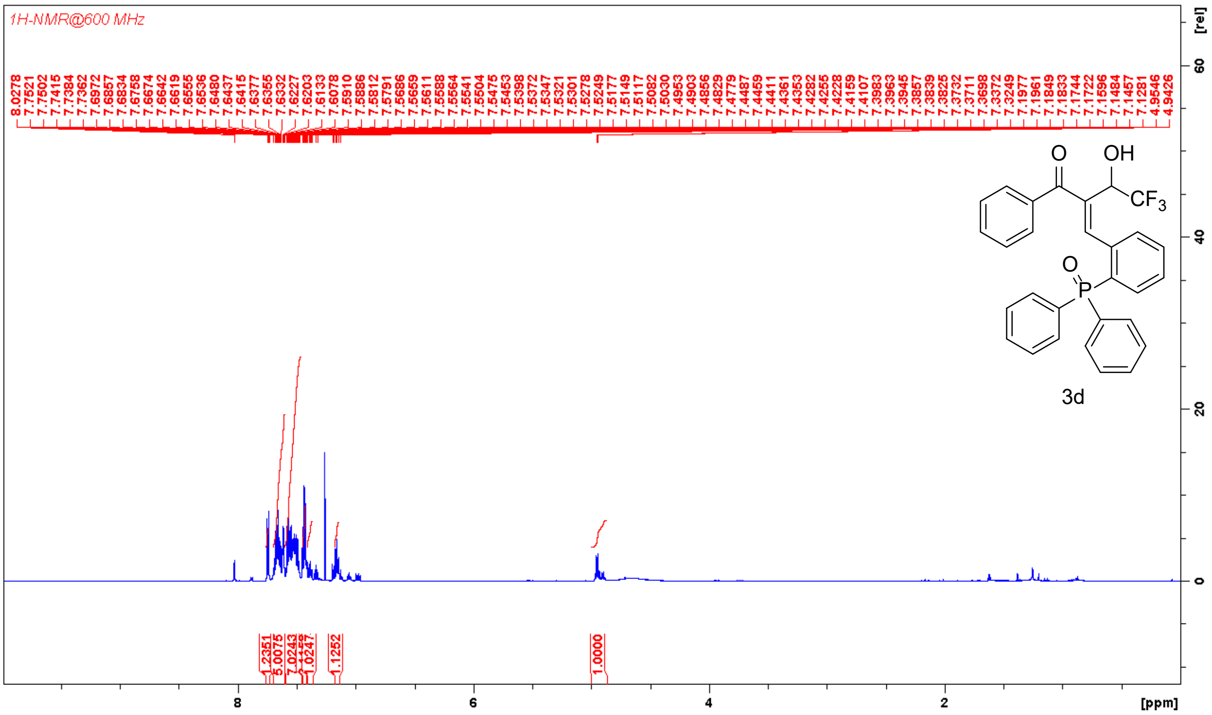

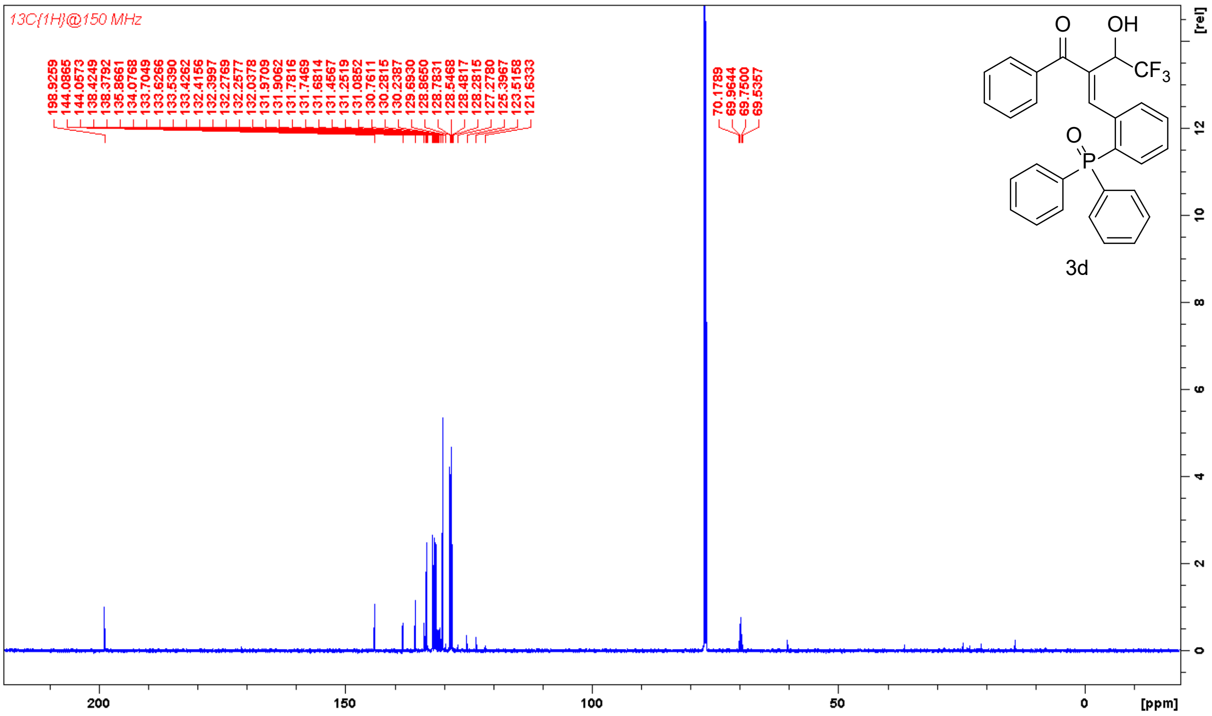
13C{1H}@150 MHz
198.9259
144.0865
144.0573
138.4249
138.3792
135.8661
134.0768
133.7049
133.6266
133.5390
133.4262
132.4156
132.3997
132.2769
132.2577
132.0378
131.9709
131.9062
131.7816
131.7469
131.6814
131.4567
131.2519
131.0852
130.7611
130.2815
130.2387
129.6930
128.8650
128.7831
128.5468
128.4817
128.2815
127.2780
125.3967
123.5158
121.6333
70.1789
69.9644
69.7500
69.5357
O
OH
CF3
O
P
3d
[rel]
[ppm]

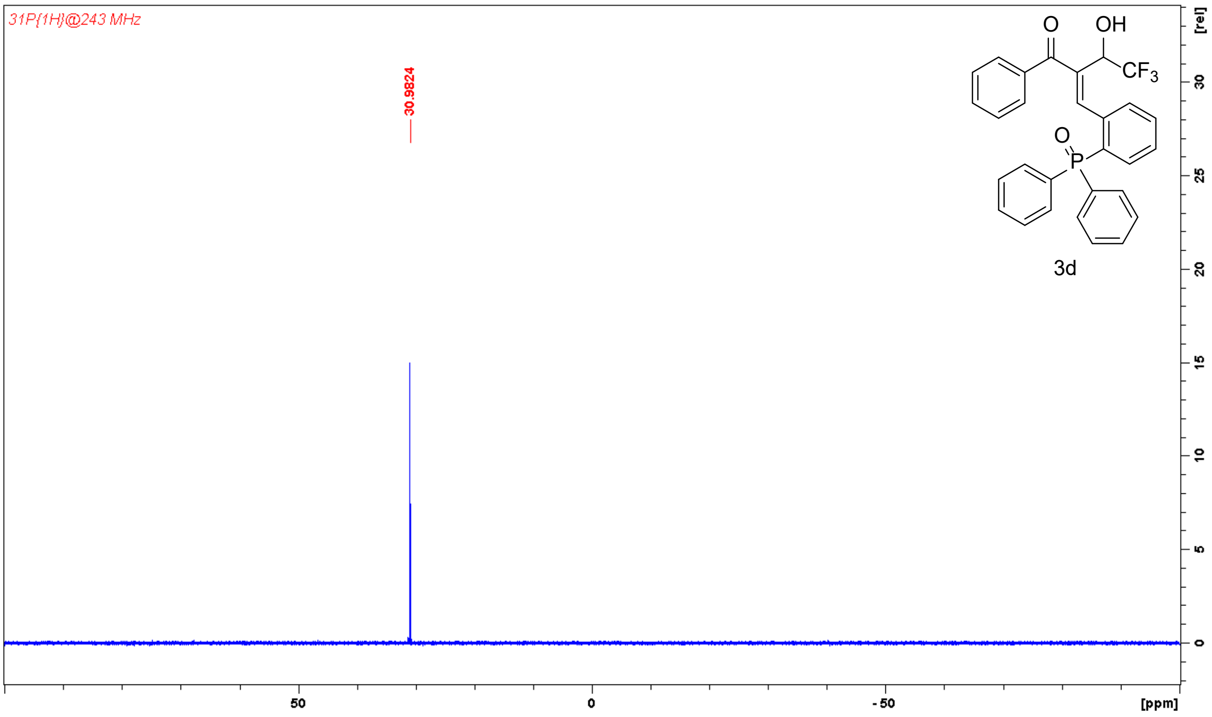
31P{1H}@243 MHz
30.9824
O
OH
CF3
O
P
3d
[rel]
[ppm]

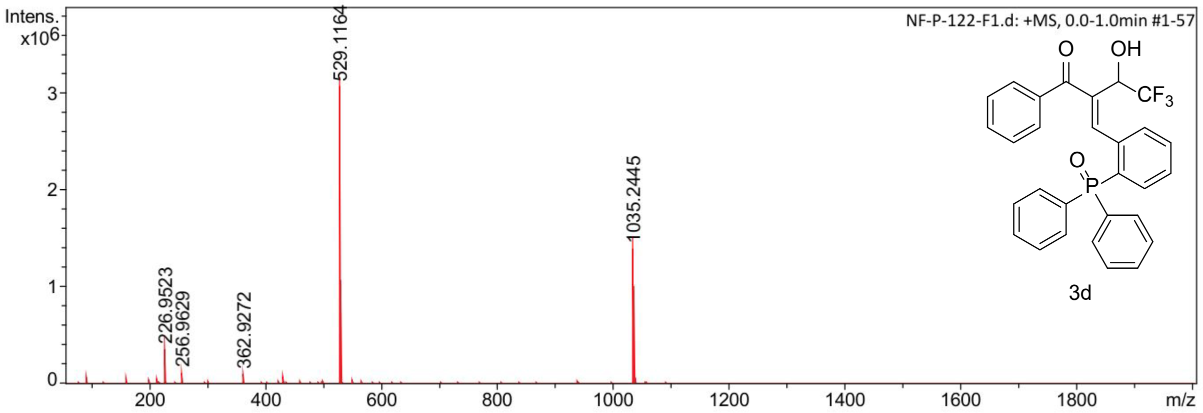
Intens.
x10^6
3
2
1
0
529.1164
1035.2445
226.9523
256.9629
362.9272
200
400
600
800
1000
1200
1400
1600
1800
m/z
NF-P-122-F1.d: +MS, 0.0-1.0min #1-57
O
OH
CF3
O
P
3d

Spectra of 6:

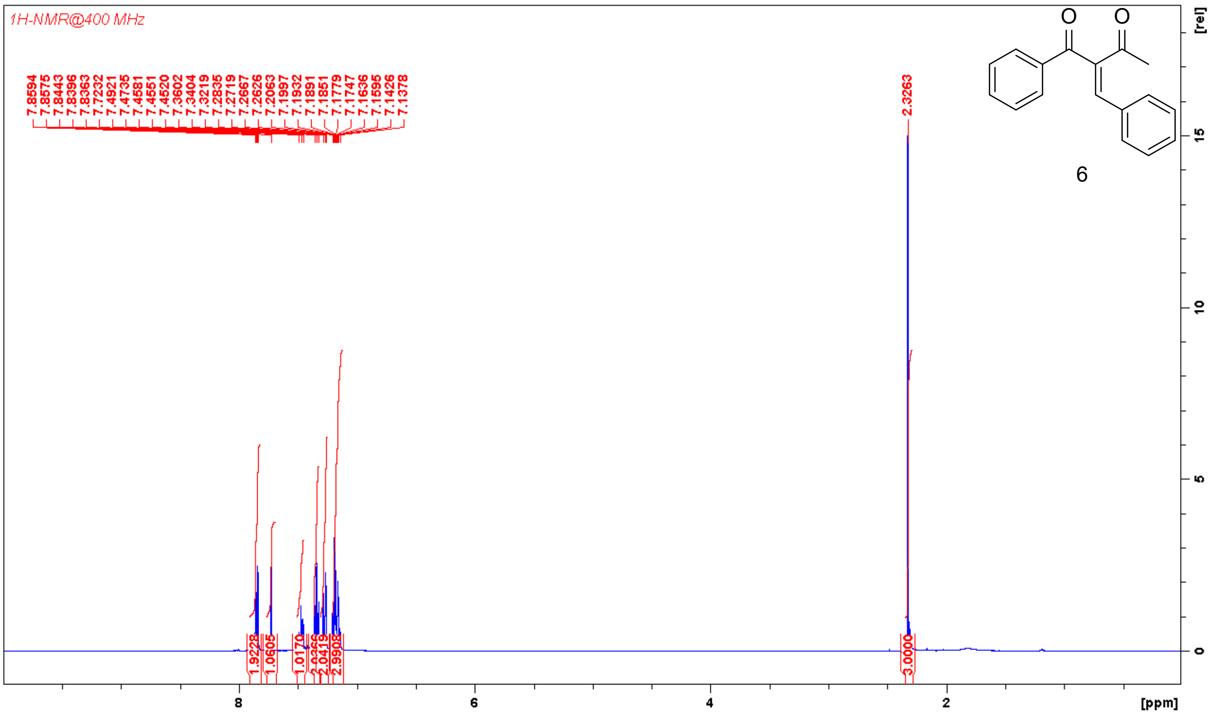
1H-NMR@400 MHz
6
[ppm]
[rel]

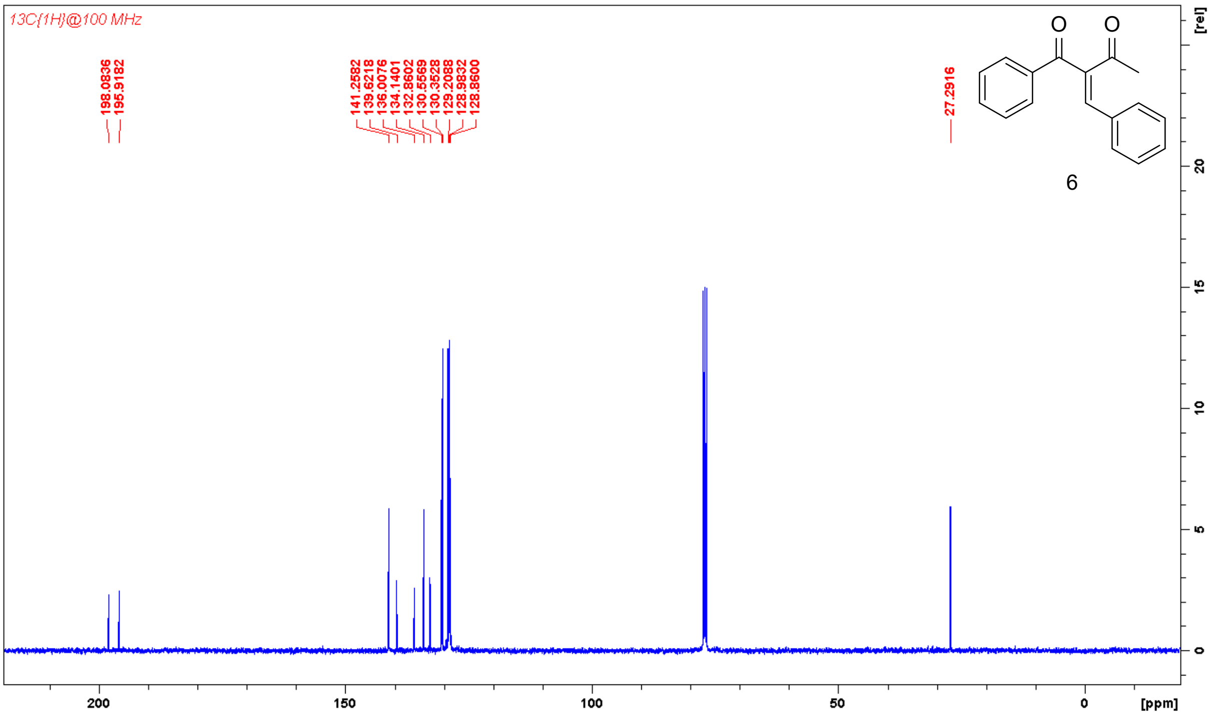
13C{1H}@100 MHz
198.0836
195.9182
141.2582
139.6218
136.0076
134.1401
132.8602
130.5569
130.3528
129.2088
128.9832
128.8600
27.2916
6
[ppm]
[rel]

Spectra of 7:

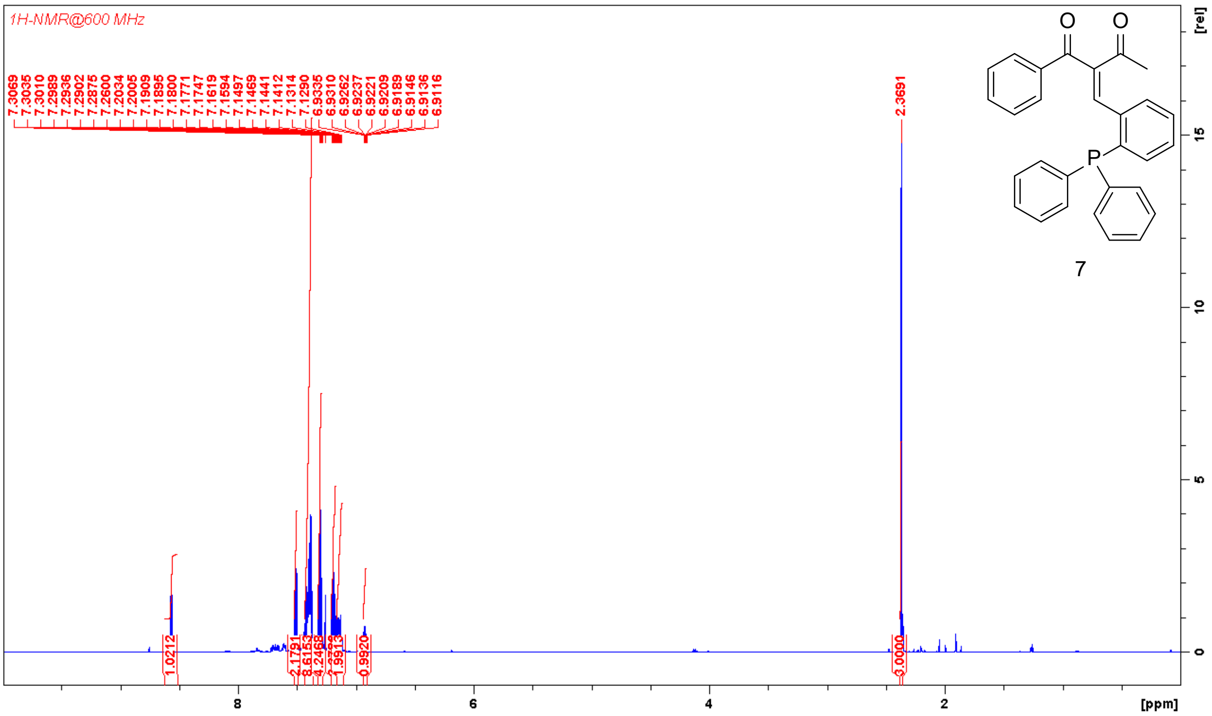

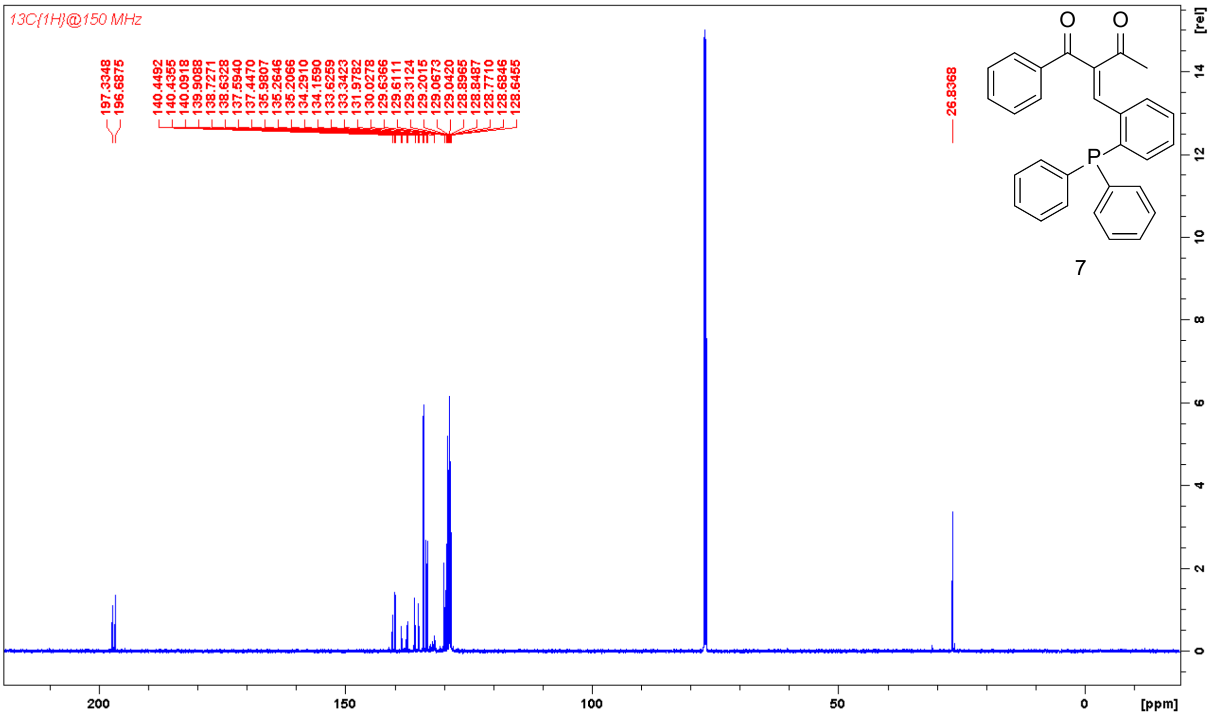

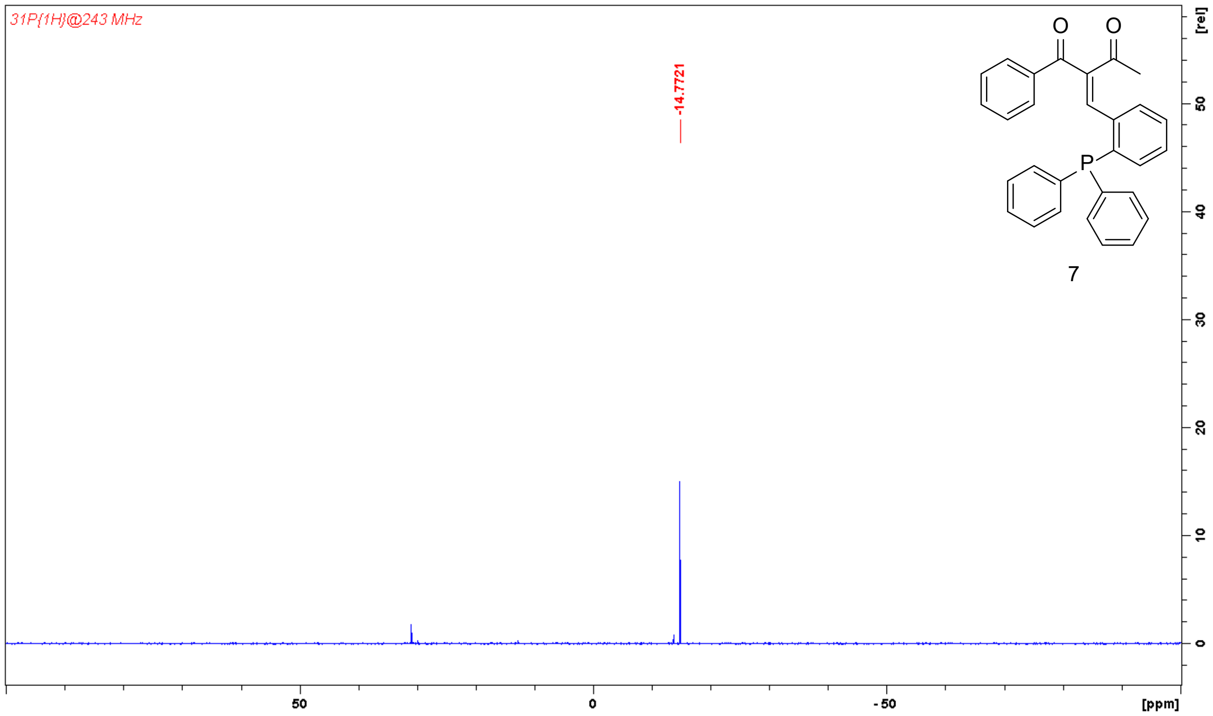
31P{1H}@243 MHz
-14.7721
O
O
P
7
[rel]
50
40
30
20
10
0
50
0
- 50
[ppm]

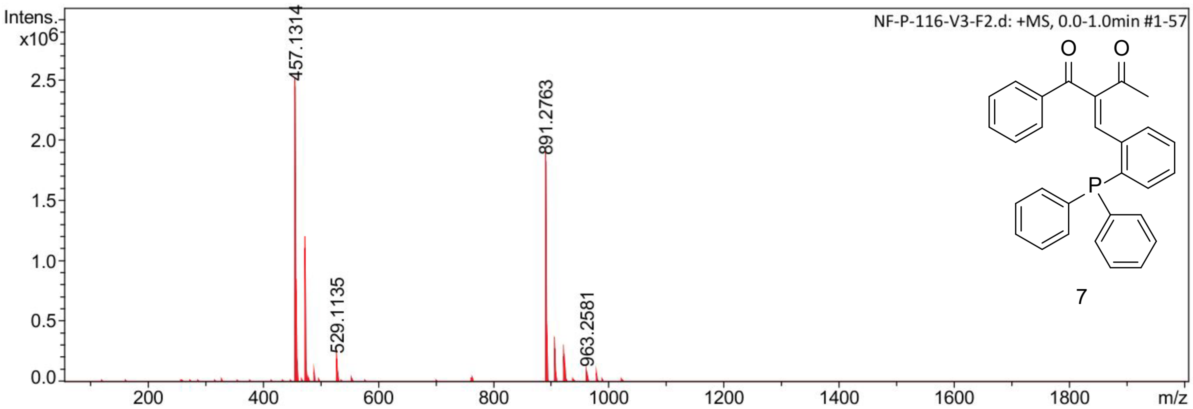
Intens.
x10^6
2.5
2.0
1.5
1.0
0.5
0.0
457.1314
529.1135
891.2763
963.2581
NF-P-116-V3-F2.d: +MS, 0.0-1.0min #1-57
O
O
P
7
200
400
600
800
1000
1200
1400
1600
1800
m/z
